# Mitoclone2: an R package for elucidating clonal structure in single-cell RNA-sequencing data using mitochondrial variants

**DOI:** 10.1093/nargab/lqae095

**Published:** 2024-08-09

**Authors:** Benjamin Story, Lars Velten, Gregor Mönke, Ahrmad Annan, Lars Steinmetz

**Affiliations:** Genome Biology Unit, European Molecular Biology Laboratory (EMBL), Heidelberg, Germany; Department of Biosystems Science and Engineering, ETH Zurich, Basel, Switzerland; Center for Genomic Regulation (CRG), The Barcelona Institute of Science and Technology, Barcelona, Spain; Developmental Biology Unit, European Molecular Biology Laboratory (EMBL), Heidelberg, Germany; Department of Computational Biology, University of Lausanne, Lausanne, Switzerland; Genome Biology Unit, European Molecular Biology Laboratory (EMBL), Heidelberg, Germany; Department of Genetics, Stanford University School of Medicine, Stanford, CA, USA; Stanford Genome Technology Center, Palo Alto, CA, USA

## Abstract

Clonal cell population dynamics play a critical role in both disease and development. Due to high mitochondrial mutation rates under both healthy and diseased conditions, mitochondrial genomic variability is a particularly useful resource in facilitating the identification of clonal population structure. Here we present mitoClone2, an all-inclusive R package allowing for the identification of clonal populations through integration of mitochondrial heteroplasmic variants discovered from single-cell sequencing experiments. Our package streamlines the investigation of this phenomenon by providing: built-in compatibility with commonly used tools for the delineation of clonal structure, the ability to directly use multiplexed BAM files as input, annotations for both human and mouse mitochondrial genomes, and helper functions for calling, filtering, clustering, and visualizing variants.

## Introduction

Genetic variability between clonal populations within an individual presentation of cancer, termed intratumor heterogeneity (IH), is a hallmark of most cancers and considered a major driver of cancer progression and relapse (1). Similarly, studies in healthy tissues aimed at lineage tracing in the context of development can also benefit from the ability to distinguish between the progeny of different founder cells. A historical genomics hurdle in identifying intra-sample genetic variation was the difficulty in distinguishing coexisting clonal populations from bulk sequencing data. With the advent of high-throughput single-cell sequencing technologies, scientists are now better able to capture and delineate cell-to-cell variability across -omics technologies.

In the context of cancer, a quantitative analysis of IH allows for the creation of cancer phylogenetic trees (CPTs) which trace the path of a tumor as it evolves from healthy cells. Uncovering the key steps involved in this pathogenic transformation is essential to understanding cancer initiation/relapse and can expose genetic changes that are vulnerable to therapeutic intervention. Furthermore, beyond applications in disease, the ability to track cells as they proliferate and differentiate is of great relevance to the field of developmental biology given that most methods for lineage tracing are costly and invasive (2). Using endogenous mitochondrial genetic variability, which is easily accessible, may help overcome these obstacles and provide a clearer picture of phenomena such as stem cell proliferation and tissue regeneration.

Despite the abundance of sequencing technologies, methods that computationally mine single-cell datasets for the presence of somatic nuclear variants are plagued by a lack of coverage and the noise inherent to single-cell sequencing. And although methods exist for genotyping single cells at sites of interest, doing so in tandem with molecular profiling methods such as RNA-seq is challenging, especially in scenarios marked by a low mutational burden within the nucleus. However, recent studies have shown that mitochondrial reads are a potential treasure trove for uncovering clone-specific mutations (3,4). The mutation rate of the mitochondrial genome is drastically elevated compared to the nuclear genome and the RNA coverage in single-cell sequencing experiments is high (5).

Capitalizing on this observation, we describe a new package, providing an all-in-one R library for the computational investigation and detection of intercellular heterogeneity with a focus on mitochondrial variants extracted from single-cell RNA-sequencing (scRNA-seq). Our package builds on existing methods including an initial prototype (6) and deepSNV (7). We enable biologists with limited programming experience to detect intercellular heterogeneity from mitochondrial reads in their single-cell datasets, all within R. We expand beyond the original mitoClone package by providing extra functionality including fast variant extraction from BAM files containing multiple single cells (i.e., multiplexed), out-of-the-box functionality (i.e., no external dependencies), extended compatibility with both the mouse mm10 and human hg19 genomes, and access to more CPT-building tools (8). mitoClone2 is now optimized for easy integration into bioinformatic pipelines. Finally, our approach matches or exceeds alternative methods in terms of performance and offers a level of portability that is not found in other software packages (Supplementary Figure S1).

## Materials and methods

**Figure 1.**
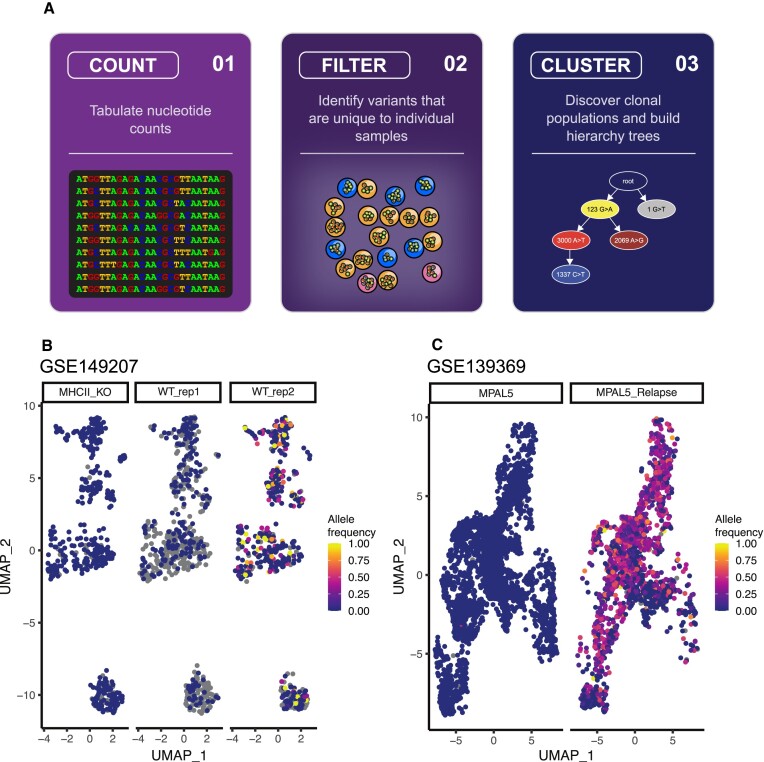
Undetected clonal variants identified in public datasets by applying mitoClone2. (**A**) Illustration of the mitoClone2 workflow. (**B**) UMAP of cells from a single-cell RNA-seq experiment conducted on mouse thymuses, focusing on three mice (one MHCII knock-out mouse and two wild-type mice) that share a consistent C57BL/6 genetic background. Color represents allele frequency of the chrM:13575 T>C mutation which marks a clonal population of cells. (**C**) UMAP of cells from a single-cell RNA-seq experiment of a mixed-phenotype acute leukemia patient showing acquisition of a novel clonal population following cancer relapse. Color represents allele frequency of the chrM:15596 G>C mutation. All gray points represent cells with insufficient coverage to call a variant.

mitoClone2 quantifies mitochondrial and nuclear variants directly from input BAM files. The package accommodates both individual BAM files per cell or multiplexed BAM files (e.g., 10× Genomics Chromium/Visium). Variants of interest are identified through two possible methods. Common to both is that variants present within the population must meet certain quality thresholds that are pre-defined by the user. These include the coverage over specific variant sites, the proportion of the overall and discrete populations allowed to exhibit a variant, the minimum variant allele frequency, and the underlying depth and read-quality of bases at variant sites (Figure 1A).

The first method is used in cases where data from multiple individuals/samples (e.g. different patients or cohorts) are available. All variants are extracted and then filtered to identify mutations unique to individuals. In such cases false-positive events due to biological (e.g. RNA-editing) or technical (e.g. PCR bias) noise can be excluded. The second method uses an exclusion list which is necessary when only a single sample is available. In this case, a database of problematic variants is provided that allows for the exclusion of sites that are in low-complexity/repeat regions, known RNA-editing sites, or arising due to re-occurring methodological errors (e.g., due to specific aligners or sequencing technologies) (9). Further information on both filtering methods can be found in the tutorial provided in the Supplementary Section (Supplementary Figures S3–S8).

After either of these filtering processes, what remains are candidate variants underlying biological differences (i.e. clonal populations). For cancer samples, we generate matrices of variant genotypes for compatibility with commonly used tools for generating CPTs (8,10). Additionally, instructions are provided for transferring metadata to other commonly-used packages for single-cell analysis, such as Seurat (11).

## Results

To illustrate the functionality of our package, we investigated the presence of mitochondrial variants in two published scRNA-seq datasets. The first was a study of the mouse thymus using Smart-seq2 and SMARTer single-cell RNA sequencing data, GEO accession: GSE149207 (12). The second included 10× Genomics 3′ single-cell RNA sequencing data at multiple time-points from human mixed phenotype leukemia patients, GEO accession: GSE139369 (13). The variants discovered in both cases were, up until now, unpublished (Figure 1B, C, Supplementary Figure S2). Our demonstration shows that by using mitoClone2, mitochondrial variants demarcating clonal populations are readily identified from scRNA-seq data across species. In the cancer context, we are able to detect novel clonal populations after cancer relapse.

## Discussion

The mitoClone2 package enables the identification of clonal populations with ease by harnessing mitochondrial variants detected using single-cell sequencing technology. Furthermore, by providing a tool that works with scRNA-seq, we allow researchers to retain the ability to profile the transcriptome while simultaneously collecting genotype information (6). Our package makes elucidating clonal structure from any single-cell dataset straightforward and paves the way for downstream characterization of clonal cell populations such as through differential gene expression testing or clinical profiling. The method is applicable not only to other cancer datasets but also to other diseases and model systems.

## Supplementary Material

lqae095_Supplemental_File

## Data Availability

The release-version of the software is freely available to install directly in R and is hosted by Bioconductor: https://bioconductor.org/packages/release/bioc/html/mitoClone2.html. The code for all present and past versions is also hosted freely by Bioconductor: https://code.bioconductor.org/browse/mitoClone2/. The developmental version is available at: https://github.com/benstory/mitoClone2. Code for the new supplementary section is available at: https://github.com/benstory/mitoClone2_supplemental.
