# Supplementary material for: Mitoclone2: an R package for elucidating clonal structure in single-cell RNA-sequencing data using mitochondrial variants
Source: NAR Genom Bioinform. 2024 Aug 9;6(3):lqae095. doi: 10.1093/nargab/lqae095 (PMC11310777; doi:10.1093/nargab/lqae095)
Supplement: lqae095_Supplemental_File [file lqae095_supplemental_file.pdf]

mitoClone2

Supplemental Information

Methods

All benchmarking was done in a CentOS Linux 7 server environment with access to two AMD EPYC 7601 32-Core Processors and approximately 1.96 TB of RAM. In all cases where possible 30 processing threads were used. All non-10x human BAM files were generated in an identical fashion; reads were aligned using STAR v2.7.2b (Dobin *et al.*, 2013) to the UCSC Genome Browser's hg38 assembly with transcript annotations from Ensembl release 95. All 10x BAM files were downloaded directly from the NCBI Sequence Read Archive.

Benchmarking was undertaken for several state-of-the-art variant calling tools: GATK v4.0.9.0 (McKenna *et al.*, 2010), bcftools v1.9 (Li, 2011), and freeBayes v1.3.4 (Garrison and Marth, 2012), which were developed primarily for bulk sequencing data, as well as mgatk v0.6.3 (Lareau *et al.*, 2021) and MQuad v0.1.4 (Kwok *et al.*, 2022), which are specifically designed for profiling mitochondria in single-cell sequencing datasets. Tools were only included in a given comparison wherein they had built-in capability to perform the specific task. In each case nucleotide tabulation and variant calling was performed across the entire mitochondrial genome.

We evaluated the different software packages for their ability to extract mitochondrial variants, using three specific metrics: 1) the speed of tabulating nucleotide counts across the mitochondrial genome from single cells, 2) the rapidity in calling variants from these nucleotide count tables, and 3) the true positive rate and false positive rate of each tool in identifying bona fide mitochondrial variants (Supp. Fig. 1). This latter comparison included two patients each exhibiting multiple clonal cell populations, marked by mitochondrial mutations that were verified using orthogonal DNA sequencing.

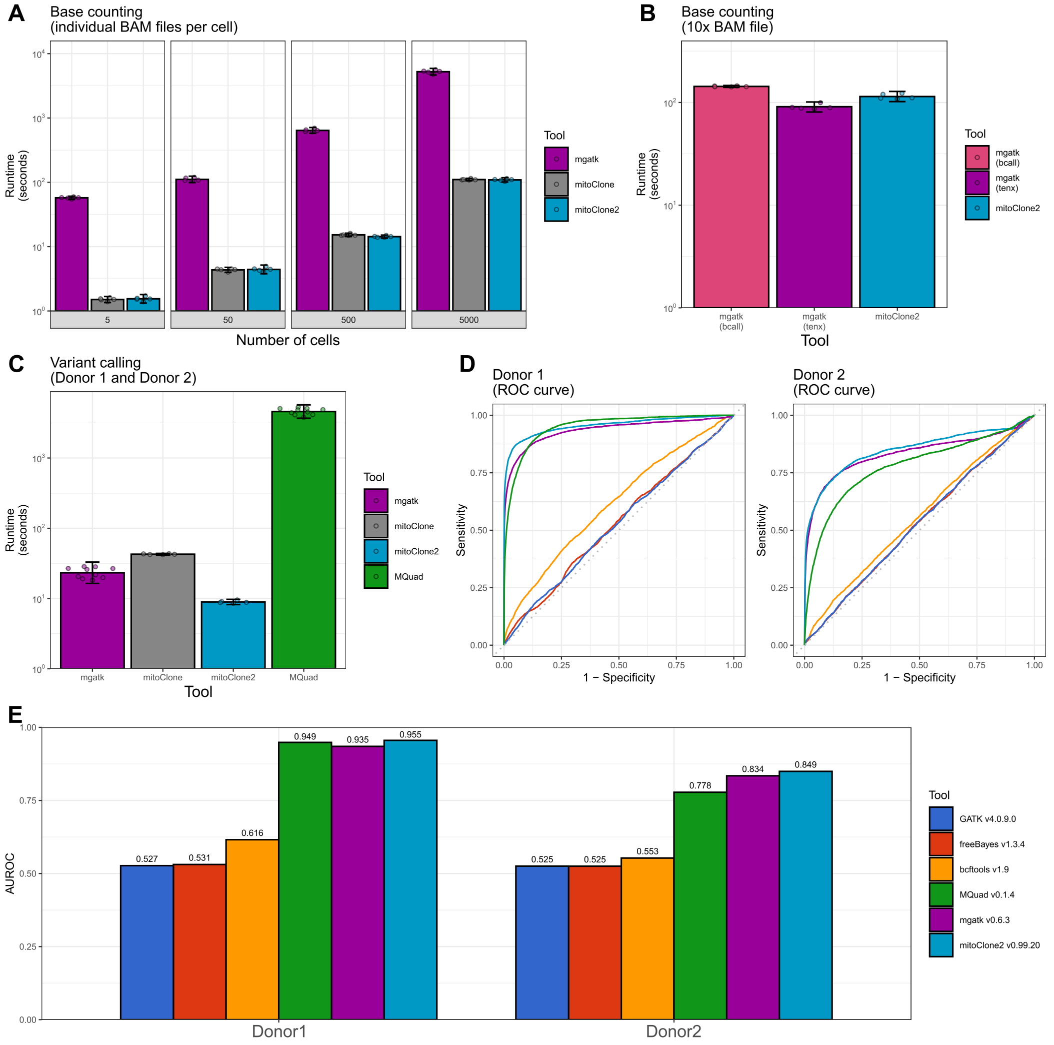

Supplementary Figure S1. mitoClone2 compared with other existing methods. **(A)** Runtime comparison for counting nucleotides across the mitochondrial genome from 5, 50, 500, and 5000 cells using individual BAM files for each cell. Sequencing data obtained from a previous publication (Velten *et al.*, 2021; EGA accession: EGAS00001003414). **(B)** Runtime for counting nucleotides from multiplexed cells in a single 10x Chromium scRNA-seq BAM file (Granja *et al.*, 2019; GEO accession: GSE139369). Tested mgatk modes include *bcall* (barcode agnostic) and *tenx* (using prior knowledge of barcodes post-filtering). **(C)** Runtime for calling mitochondrial variants using precomputed nucleotide counts from an existing publication (Ludwig *et al.*, 2019; GEO accession: GSE115214). For panels A through C, replicates are represented by dots and error bars denote 2 standard deviations about the mean. **(D)** Receiver operating characteristic (ROC) using a pairwise similarity metric for mitochondrial variants derived from verified clonal cell populations. Based on variant calls from panel C. **(E)** Area under the ROC (AUROC) performance metric for the results of multiple variant callers applied to the two donor samples from panel D. AUROC/ROC calculations were adapted from a previously used method (Lareau *et al.*, 2021). Color legend (bottom-right) applies to panels D and E.

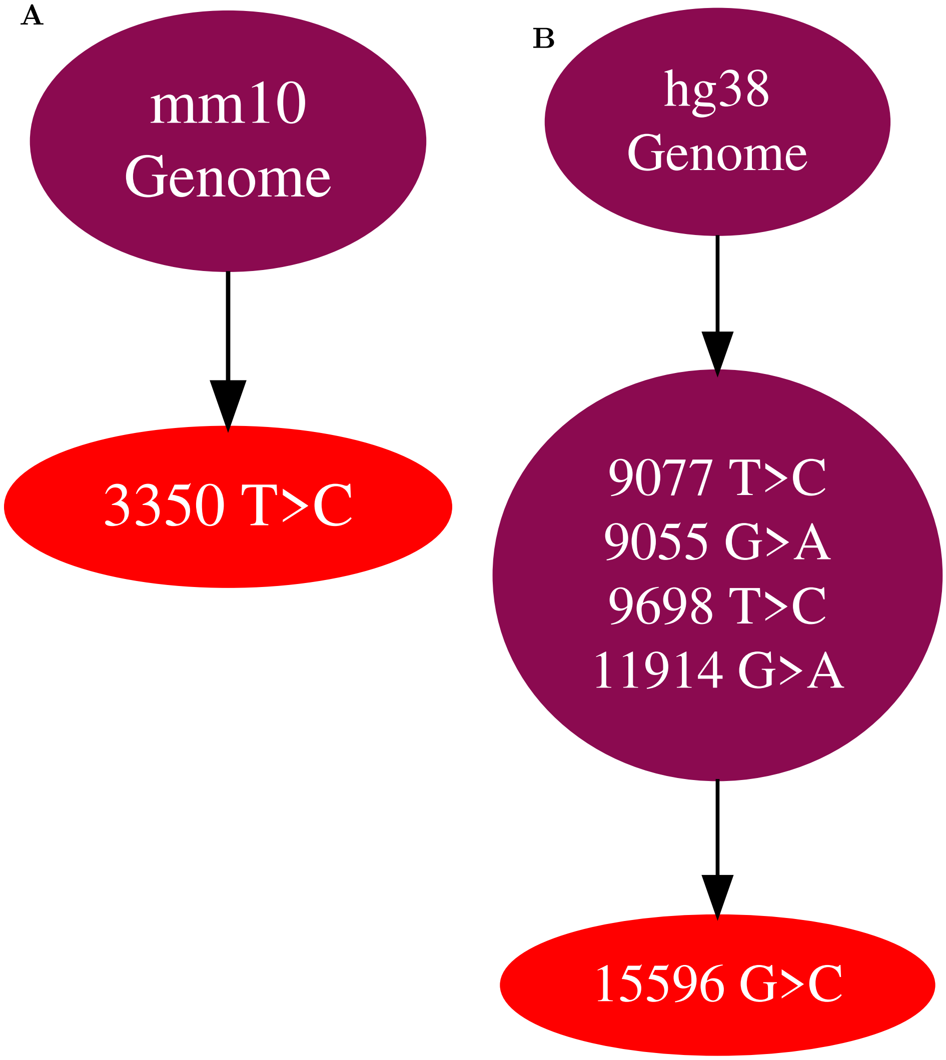

Supplementary Figure S2. Mutation trees for mouse and human datasets. **(A)** Mutation tree depicting the variants present in WT_rep2 from dataset GSE149207. **(B)** Mutation tree illustrating the variants present in patient MPAL5 after disease relapse from dataset GSE139369. Reference variants are indicated in purple, representing reference alleles in the genome and non-reference alleles shared by the population (e.g. all mice or the individual MPAL5 patient), while novel variants in subclones are highlighted in red.

mitoClone2: Supplementary Tutorial

Benjamin Story, Ahrmad Annan

13 June 2024

Contents

References **1**

1 Demonstration of mitochondrial variant discovery from RNA-sequencing data **2**

2 Complete workflow **3**

2.1 Load packages . . . 3

2.2 Prepare some helper functions . . . 3

2.3 Call variants from the RNA-seq data . . . 4

2.4 Examine the excluded universally shared variants . . . 4

2.5 Extract putative private variants . . . 4

2.6 Prepare metadata for the downstream plotting . . . 5

2.7 Investigate allele frequencies of putative private variants across sequencing technologies . . . 5

2.8 Verifying consistency between ATAC-seq and RNA-seq for putative private variants . . . 6

2.9 Identify variants resulting from cancer evolution . . . 9

3 Alternate workflow with the *Exclusionlist* filtering method **11**

3.1 Call variants from the RNA-seq data using provided *Exclusionlists* . . . 12

3.2 Downstream analysis similar to 2.5-2.9 . . . 12

3.3 The *Exclusionlist* filtering method compared to the *Cohort* method . . . 14

4 Adding metadata to Seurat objects **15**

References

Corces, M., Buenrostro, J., Wu, B. *et al.* Lineage-specific and single-cell chromatin accessibility charts human hematopoiesis and leukemia evolution. *Nat Genet* **48**, 1193–1203 (2016). https://doi.org/10.1038/ng.3646. PMID: 27526324

1 Demonstration of mitochondrial variant discovery from RNA-sequencing data

As a proof of concept, we demonstrate the ability of the mitoClone2 package to extract *bona fide* private mutations from paired bulk ATAC-seq and RNA-seq samples. The goal here was to verify that variants in the mtDNA, which are specific to individual populations of cells, can be readily detected using RNA-seq data alone. We downloaded public bulk ATAC-seq and RNA-seq experimental data which were performed in parallel on cancer patients.

The data used for this analysis, from Corces *et al.* (cited in **References**), can be downloaded from the NCBI Gene Expression Omnibus (GEO) which is accessible at: https://www.ncbi.nlm.nih.gov/geo/query/acc.cgi?acc=GSE75384

The RNA-seq reads were aligned to the *Homo sapiens* hg38 genome (Ensembl release 95) using `STAR v2.7.2b` with default parameters. The ATAC-seq reads were trimmed and aligned to the *Homo sapiens* hg38 genome using `bowtie2 v2.3.0` with the following parameters: `-X 2000`.

Count tables for each sample were tabulated using the `baseCountsFromBamList` a function included in the mitoClone2 package. The sample name for each putative patient was inferred from the GEO metadata. Only the following patients were included: `6792, 1022, 2596, 5483, 6926, 7256, SU048, SU070, SU209, SU351, SU353, SU444, SU484, SU496, SU501, SU575, SU583, and SU654` as these patients had both DNA (i.e. ATAC-seq) and RNA data available.

We note here that although this is bulk data, we can still treat each sample as if it were a single cell.

We note at this point that there are two possible filtering methods that can be used for downstream analysis.

1.0.1 *Cohort* Method:

- Preferred for controlling wet-lab variability by using multiple independent single-cell datasets, generated using the same sequencing method, to exclude shared variants and identify patient/time-point specific variants.
- The excluded variants could be biological (common polymorphisms) or technical (artifacts from specific reagents or bioinformatics tools).
- This method helps filter out both technical and biological noise, by determining what signal can be attributed to background noise.

1.0.2 ***Exclusionlist*** Method:

- Utilizes established lists of known artifactual or challenging genomic regions, encompassing three sets:
  - Known RNA-editing sites in mitochondria from REDIportal (PMID: 27587585)
  - Regions flanking homopolymer stretches of 3 or more nucleotides
  - Shared variants specific to a given sequencing method
- The latter set of shared variants, included in the package, is derived from datasets generated using a modified Smart-seq2.
- This method is ideal for users who lack access to multiple datasets or require a fast, predefined filtering option.

The following sections provide a detailed walkthrough of both filtering methods, starting with the *Cohort* method.

2 Complete workflow

2.1 Load packages

```
library(mitoClone2)
library(reshape2)
library(viridis)
library(ComplexHeatmap)
library(ggplot2)
```

2.2 Prepare some helper functions

A variety of extra functions are provided to minimize code redundancy.

```
#-- Function that converts variant string format from 'X13370.G' to '1337 0>G'
fixvarName <- function(x) {
    gsub("\\.", ">", gsub("^X", "", x))
}

#-- Function that takes a dataset and name and gets mean allele freq. per patient
getptMean <- function(Data, name) {
    df <- data.frame(t(Data))
    df.l <- lapply(split(df, gsub("\\-.*", "", row.names(df))), colMeans)
    df.l <- melt(do.call(cbind, df.l))
    colnames(df.l) <- c("var", "Patient", name)
    return(df.l)
}
```

This vignette uses data that is large (~30 MB) and thus not included with the package, but it can be downloaded separately via GitHub at: https://github.com/benstory/mitoClone2_supplemental.

The **mitoClone2** package provides some sample datasets. However, to emphasize the ability of mitoClone2 to detect real mitochondrial variants in RNA-seq data alone, we opted to use an additional more appropriate dataset where orthogonal variant confirmation via ATAC-seq was possible. These nucleotide count tables, imported below, were generated by the `baseCountsFromBamList` function - for ATAC-seq the `bam2R` quality threshold was set to 30 instead of the default.

```
#-- Here is an example of how we would generate our allele count files locally - SKIPPED
## vars.rna <- baseCountsFromBamList(bamfiles =
## list.files('corces_RNA','\\.bam$',full = TRUE), sites =
## 'chrM:1-15000') the files need to be located in R's working
## directory!
#-- Download the external data from the mitoClone2 Supplemental GitHub Page
SuppGitURL <- "https://github.com/benstory/mitoClone2_supplemental/raw/main/"
## system(paste0('wget -nv ',
## SuppGitURL,'corces_nt_counts_per_position_rna.RDS'))
## system(paste0('wget -nv ',
## SuppGitURL,'corces_nt_counts_per_position_quality30_atac.RDS'))
#-- Loading the external data from GSE74246 (rna)
vars.rna <- readRDS("corces_nt_counts_per_position_rna.RDS")
#-- Loading the external data from GSE74912 (atac)
vars.atac <- readRDS("corces_nt_counts_per_position_quality30_atac.RDS")
```

We run mutationCallsFromCohort on the RNA-seq allele count tables for each of these patient samples using default parameters excepting for `MINFRAC.OTHER=0.2`, `MINREADS=500`, `MINCELL=1`, `MINCELLS.PATIENT=1`.These parameters are modified from the default values because we are not dealing with single-cell data. The `MINCELL` parameter now refers to the total number of samples from each individual patient. Given that there are two or more samples per patient, we require that the variants be present in at least a single sample.

The resulting 153 variants from this command were further filtered to remove 8 universal variants with an allele frequency over 5% in more than 6 RNA-seq samples (i.e. non-patient exclusive mutations) and variants that clustered together within a 3-bp window, leaving 145 putative variants of interest.

2.3 Call variants from the RNA-seq data

```
vars.rna.call <- mutationCallsFromCohort(vars.rna, MINFRAC = 0.1, MINFRAC.PATIENT = 0.01,
    MINFRAC.OTHER = 0.2, MINREADS = 500, MINCELL = 1, MINCELLS.PATIENT = 1,
    patient = gsub("\\-.*", "", names(vars.rna)), genome = "hg38", sites = "chrM:1-16569",
    USE.REFERENCE = TRUE)
```

2.4 Examine the excluded universally shared variants

```
#-- We extract the list of excluded sites and save for downstream analysis
vars.exclusions <- vars.rna.call[["exclusionlist"]]
head(vars.exclusions)
```

```
## [1] "73 A>G"  "146 T>C" "152 T>C" "263 A>G" "646 C>T" "709 G>A"
```

```
vars.rna.call[["exclusionlist"]] <- NULL
```

2.5 Extract putative private variants

```
#-- Pull all the identified mutations that are likely exclusive to patients
candidates <- unlist(lapply(vars.rna.call, function(x) names(x@cluster)))

#-- Remove variants that appear in clusters separated by less than 3bp for each patient
candidates <- removeWindow(fixvarName(sort(unique(candidates))), window = 3)

#-- Extract counts of putative candidates from the RNA-seq
rna.counts <- pullcountsVars(vars.rna, candidates)
plotData <- rna.counts$M/(rna.counts$M + rna.counts$N)

#-- Remove variants that seem to be universal (i.e. non-patient exclusive)
#-- Our cutoff is an allele frequency over 5% in more than 6 samples
plotData <- plotData[rowSums(plotData > 0.05) <= 6, ]
print(NROW(plotData))
```

```
## [1] 145
```

2.6 Prepare metadata for the downstream plotting

Extract patient identifiers and set colors.

```
#-- Extract patient IDs
pts <- gsub("\\-.*", "", colnames(plotData))
#-- Set unique colors for each patient
pts.colors <- c("#FF7F00", "brown", "palegreen2", "khaki2", "#FB9A99",
    "darkorange4", "green1", "gold1", "steelblue4", "dodgerblue2", "darkturquoise",
    "orchid1", "black", "#6A3D9A", "blue1", "#CAB2D6", "gray70", "yellow3")
names(pts.colors) <- levels(factor(pts))
```

Prepare metadata for samples and heatmaps.

```
#-- Preparing a metadata data.frame that includes:
#-- the sample name
#-- the patient associated to the sample
#-- the technology used to generate the sample
meta.data <- melt(c(names(vars.rna), names(vars.atac)))
row.names(meta.data) <- meta.data$value
meta.data$Patient <- gsub("\\-.*", "", meta.data$value)
meta.data$Method <- paste0(gsub(".*\\_", "", meta.data$value), "-seq")
meta.data$value <- NULL
```

2.7 Investigate allele frequencies of putative private variants across sequencing technologies

We then extracted these same variants from both the ATAC-seq and RNA-seq (above) samples using the `pullcountsVars` function.

Prepare a heatmap of the RNA-seq data.

```
#-- Setup heatmap annotation - plotData
ha_column = HeatmapAnnotation(df = meta.data[colnames(plotData), "Patient",
    drop = FALSE], col = list(Patient = pts.colors), show_annotation_name = FALSE)
#-- Build a heatmap of the RNA-seq allele frequencies for each variant
hrna <- Heatmap(plotData, col = viridis(100, option = "plasma"), show_row_names = TRUE,
    show_column_names = FALSE, top_annotation = ha_column, column_title = "RNA-seq",
    show_column_dend = FALSE, show_row_dend = FALSE, column_title_gp = gpar(fontsize = 24),
    row_names_gp = gpar(fontsize = 2), name = "Allele\nfreq.")
```

Prepare a heatmap of the ATAC-seq data.

```
#-- Extract counts of putative candidates from the ATAC-seq
atac.counts <- pullcountsVars(vars.atac, row.names(plotData))
plotData.atac <- atac.counts$M/(atac.counts$M + atac.counts$N)

#-- Setup heatmap annotation for plotData.atac
ha_column = HeatmapAnnotation(df = meta.data[colnames(plotData.atac), "Patient",
    drop = FALSE], col = list(Patient = pts.colors))
#-- Build a heatmap of the ATAC-seq allele frequencies for each variant
hatac <- Heatmap(plotData.atac, col = viridis(100, option = "plasma"),
```

```
    show_row_names = TRUE, show_column_names = FALSE, top_annotation = ha_column,
    column_title = "ATAC-seq", show_column_dend = FALSE, show_row_dend = FALSE,
    column_title_gp = gpar(fontsize = 24), row_names_gp = gpar(fontsize = 2),
    name = "Allele\nfreq.")
```

The variants identified seem to allow clear differentiation of the individual patients across technologies.

```
#-- Plot heatmaps for both technologies side-by-side - S3
hrna + hatac
```

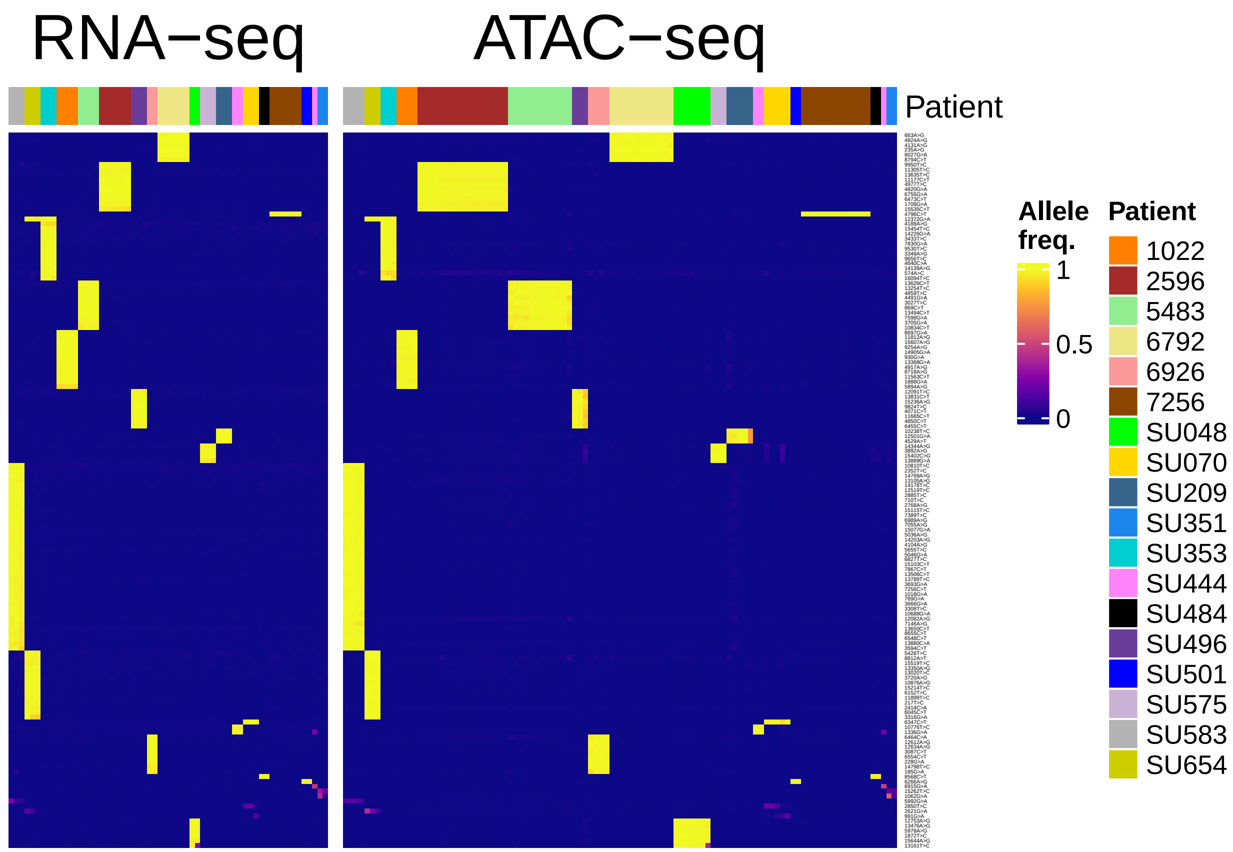

Supplementary Figure S3. Allele frequencies of mitochondrial variants identified as patient-specific from RNA-seq.

2.8 Verifying consistency between ATAC-seq and RNA-seq for putative private variants

Extract the mean allele frequencies per patient for each variant.

```
#-- Extract mean allele freq per patient per technology and merge
df.rna <- getptMean(plotData, "RNA-seq")
df.atac <- getptMean(plotData.atac, "ATAC-seq")

#-- Sanity check all data.frames should be similar
all(c(identical(df.rna$Patient, df.atac$Patient)), identical(df.rna$var,
    df.atac$var))
```

```
## [1] TRUE
```

```
#-- merge into a new data.frame
df.both <- df.rna
df.both$"ATAC-seq" <- df.atac$"ATAC-seq"

#-- Perform a correlation test on the samples with Pearson
cor_both <- cor.test(df.both$ATAC, df.both$RNA)
colnames(df.rna)[2] <- "Patient"
```

Plot the correlation between the RNA-seq and the ATAC-seq.

```
#-- Plot the correlation and allele frequency comparison - S4
ggplot(df.both, aes(x = `RNA-seq`, y = `ATAC-seq`, color = Patient)) +
    geom_point(size = 4) + theme_classic(base_size = 14) + geom_abline(slope = 1,
    color = "grey", linetype = "dashed") + scale_colour_manual(values = pts.colors) +
    annotate("text", x = 0.25, y = 0.8, label = paste0("r = ", cor_both$estimate)) +
    ylab("ATAC-seq\nAllele frequency") + xlab("RNA-seq\nAllele frequency") +
    theme(plot.margin = unit(c(1, 1, 1, 1), "cm"), legend.text = element_text(size = 8)) +
    guides(color = guide_legend(ncol = 2))
```

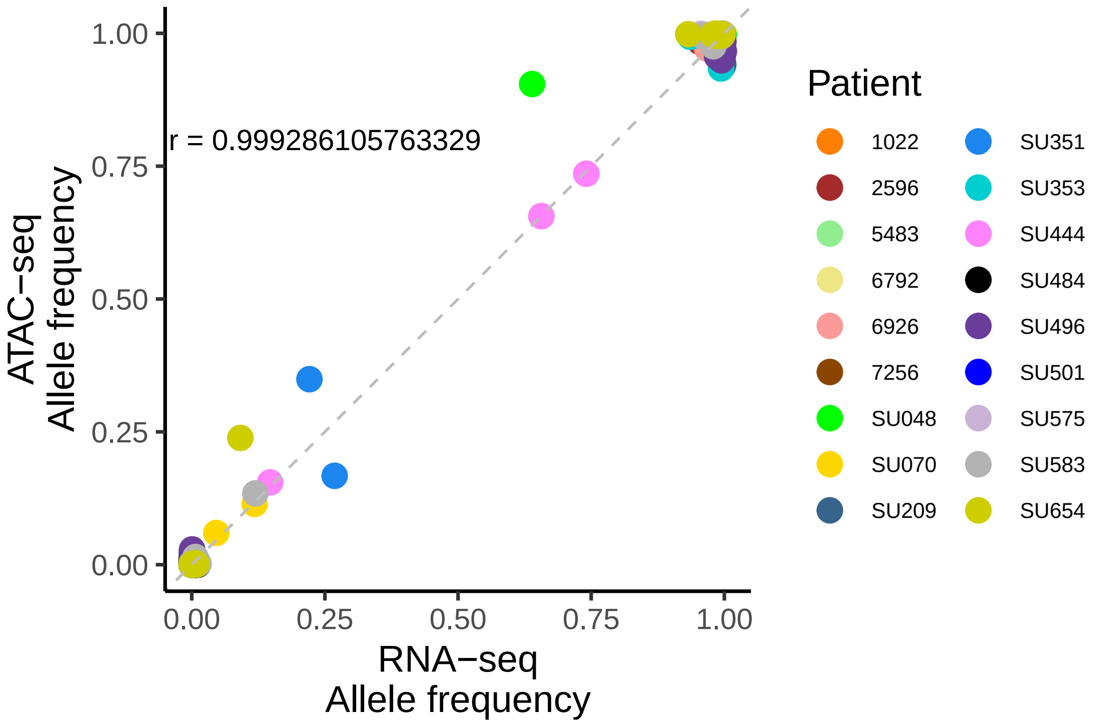

Supplementary Figure S4. Allele frequencies of RNA-seq compared to ATAC-seq with Pearson correlation coefficient.

Merge your two large allele frequency data.frames.

```r
#-- Merge the RNA-seq and ATAC-seq allele frequency data.frames
merged.vars <- merge(plotData.atac, plotData, by = "row.names", all = T)
row.names(merged.vars) <- merged.vars$Row.names
merged.vars$Row.names <- NULL
```

Calculate the Euclidean distance between samples and prepare metadata.

```r
#-- Create distance matrix from merged method data.frame
#-- The default method is set to 'euclidean'
distmat <- data.frame(as.matrix(dist(t(merged.vars))))
colnames(distmat) <- row.names(distmat)
distance.colors <- list(Method = c(`RNA-seq` = "red", `ATAC-seq` = "blue"),
    Patient = pts.colors)
ha_column <- HeatmapAnnotation(df = meta.data[colnames(distmat), ], col = distance.colors)
```

Plot the distance-matrix heatmap.

```r
#-- S5
Heatmap(distmat, col = viridis(100), show_row_names = FALSE, show_column_names = FALSE,
    top_annotation = ha_column, show_column_dend = FALSE, show_row_dend = FALSE,
    name = "Euclidean\ndistance")
```

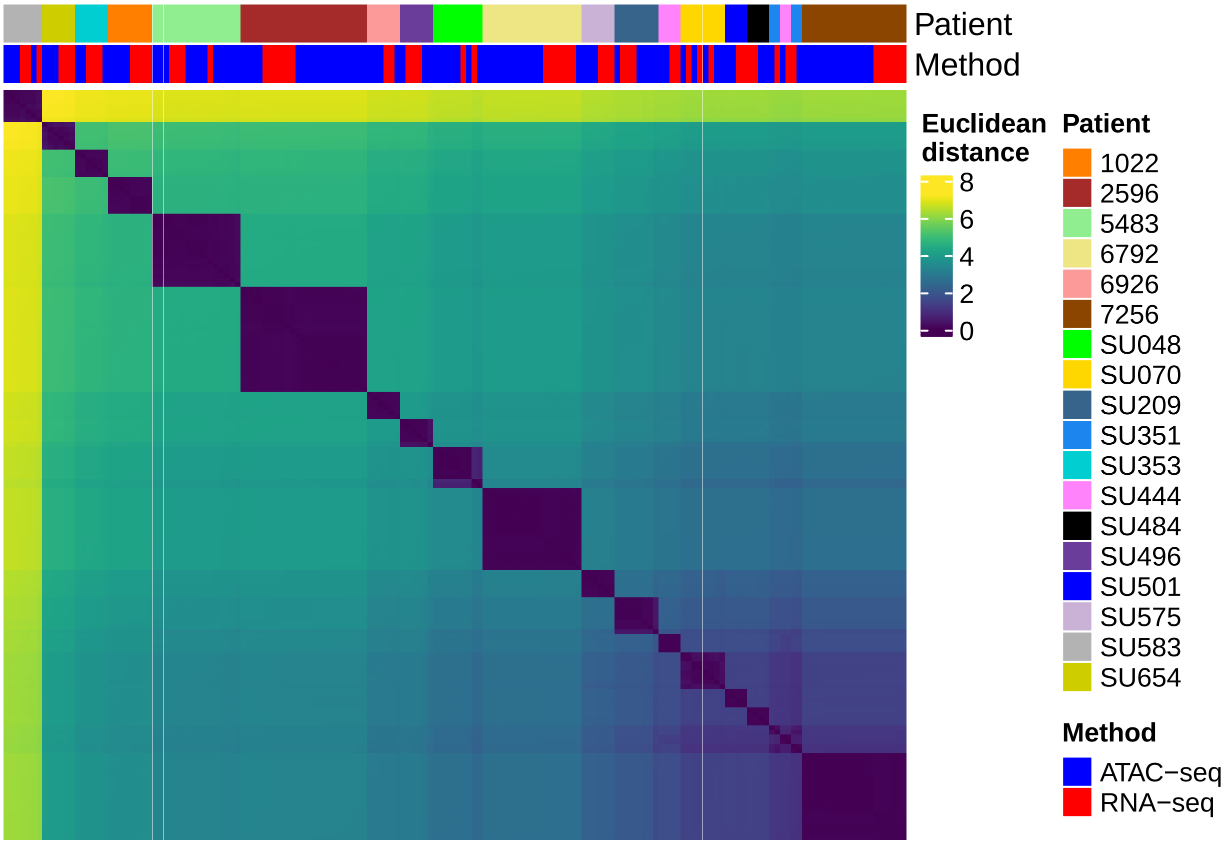

Supplementary Figure S5. Clustered distance matrix of combined variant allele frequencies from ATAC-seq and RNA-seq.

2.9 Identify variants resulting from cancer evolution

Even in this bulk sequencing experiment, we see evidence of variants that differ between the cancerous and pre-cancerous states.

Let's home in on an example, patient SU444, and extract their unique variants.

```
su444 <- fixvarName(names(vars.rna.call[["SU444"]]@cluster))
print(su444)
```

```
## [1] "1336G>A"  "6915G>A"  "10776T>C"
```

Pull the corresponding variant allele frequencies from both the ATAC-seq and RNA-seq datasets and prepare to plot.

```
#-- Extract raw AFs from the datasets
su444rna <- data.frame(plotData[su444, grep("SU444", colnames(plotData))])
su444atac <- data.frame(plotData.atac[su444, grep("SU444", colnames(plotData.atac))])

#-- Merge into a single data.frame and print
su444 <- cbind(su444rna, su444atac)
print(su444)
```

```
##          SU444.Blast_RNA SU444.LSC_RNA SU444.pHSC_RNA SU444.Leuk_ATAC
## 1336G>A     0.9960236461  0.9920427389     0.23440704    0.9984565830
## 6915G>A     0.0003128715  0.0003790751     0.44083000    0.0001405185
## 10776T>C    0.9745313053  0.9832127352     0.01153846    0.9782385060
##          SU444.LSC_ATAC SU444.pHSC_ATAC
## 1336G>A       0.9987066     0.209903122
## 6915G>A       0.0000000     0.464139344
## 10776T>C      0.9876380     0.001962709
```

```
su444_ann <- HeatmapAnnotation(df = subset(meta.data, Patient == "SU444"),
    col = list(Method = c(`RNA-seq` = "red", `ATAC-seq` = "blue"), Patient = pts.colors))
```

Plot the variant allele frequencies for this patient.

```
#-- S6
Heatmap(as.matrix(su444), col = viridis(100, option = "plasma"), show_row_names = TRUE,
    show_column_names = TRUE, top_annotation = su444_ann, column_split = rep(c("RNA-seq",
        "ATAC-seq"), each = 3), show_column_dend = FALSE, show_row_dend = FALSE,
    name = "Allele\nfreq.")
```

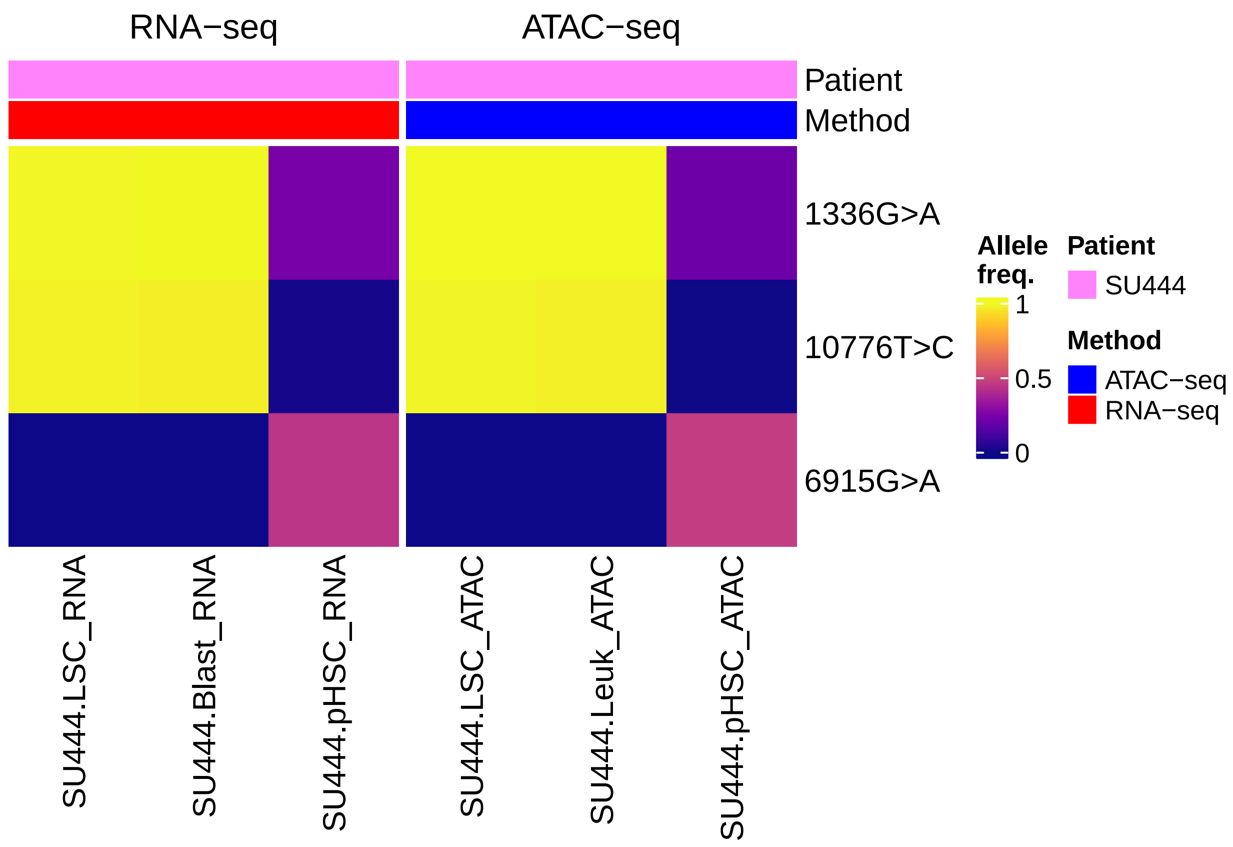

Supplementary Figure S6. A zoomed-in version of (**Supplementary Figure S3**), focused on variants specific to patient SU444.

We can also attempt to infer a cancer phylogenetic tree (CPT) relating the different mutations and illustrate their locations using the built-in `mitoPlot` function.

```
#-- If you want to reproduce the final figure (S7) you need to run this step
#-- However the figure here (below) has undergone significant post-processing
#-- Use SCITE to infer a CPT for patient SU444
clust444 <- varCluster(vars.rna.call[["SU444"]], method = "SCITE")

#-- S7 - bottom
mitoPlot(fixvarName(names(vars.rna.call[["SU444"]]@cluster))[c(1, 3, 1,
    2)], patient = rep(rev(c("pHSC", "Blast/LSC")), each = 2), showLegend = TRUE)
```

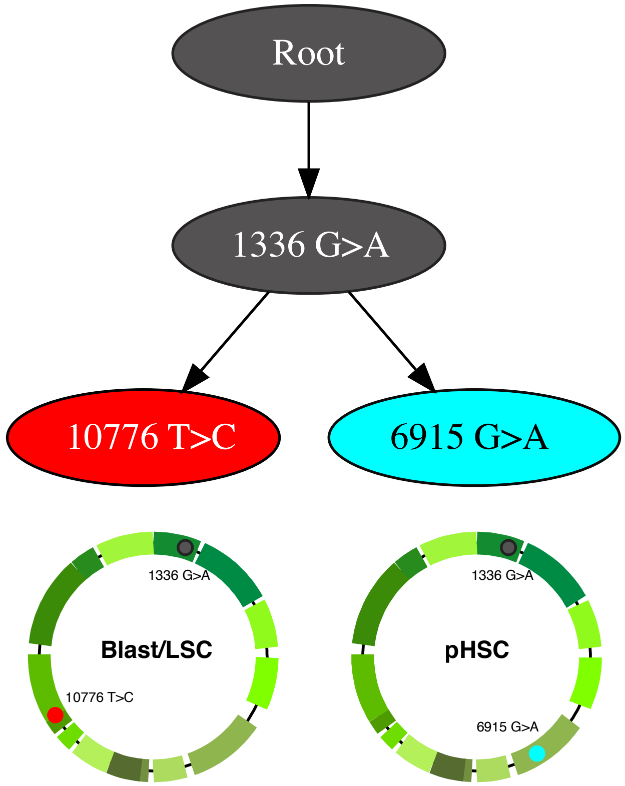

Supplementary Figure S7. The results of running `SCITE` (top) and the `mitoPlot` function (bottom) on the RNA-seq variant counts from patient SU444. The tree illustrates a potential phylogenetic relationship underlying cancerous clonal evolution differentiating the pre-leukemic (pHSC) sample from the blast/leukemic stem cell (LSC) samples.

3 Alternate workflow with the *Exclusionlist* filtering method

To complete our proof of concept, we apply a different universal variant filtering method based on existing exclusion lists that are shipped with the mitoClone2 package. This filtering method should only be used when the dataset includes only a single sample from one individual/time-point.

We re-import the allele count tables.

```
#-- Here is an example of how we would generate our allele count files locally - SKIPPED
## vars.rna <- baseCountsFromBamList(bamfiles =
## list.files('corces_RNA','\\.bam$',full = TRUE), sites =
## 'chrM:1-15000') the files need to be located in R's working
## directory!
#-- Loading the external data from GSE74246 (rna)
vars.rna <- readRDS("corces_nt_counts_per_position_rna.RDS")
#-- Loading the external data from GSE74912 (atac)
vars.atac <- readRDS("corces_nt_counts_per_position_quality30_atac.RDS")
```

We run mutationCallsFromExclusionlist on the RNA-seq allele count tables for each of these patient samples using default parameters to call mutations using the exclusion list included in the package.

3.1 Call variants from the RNA-seq data using provided *Exclusionlists*

```
# calling mutations using our exclusionlist
vars.rna.call <- mutationCallsFromExclusionlist(vars.rna)
```

```
## Subsetting for specific cells...
```

3.2 Downstream analysis similar to 2.5-2.9

From there, we will use the same downstream analysis as with the variant calling using the cohort filtering method.

```
#-- Pull all the identified mutations that are likely exclusive to patients
candidates <- sub(">", "", fixvarName(sort(unique(names(vars.rna.call@cluster)))),
    fixed = TRUE)

#-- Remove variants that appear in clusters separated by less than 3bp for each patient
candidates <- removeWindow(candidates, window = 3)

#-- Extract counts of putative candidates from the RNA-seq
rna.counts <- pullcountsVars(vars.rna, candidates)
plotData <- rna.counts$M/(rna.counts$M + rna.counts$N)

#-- Remove variants that seem to be universal (i.e. non-patient exclusive)
#-- Our cutoff is an allele frequency over 5% in more than 6 samples
plotData <- plotData[rowSums(plotData > 0.05) <= 6, ]

#-- Extract patient IDs
pts <- gsub("\\-.*", "", colnames(plotData))
#-- Set unique colors for each patient
pts.colors <- c("#FF7F00", "brown", "palegreen2", "khaki2", "#FB9A99",
    "darkorange4", "green1", "gold1", "steelblue4", "dodgerblue2", "darkturquoise",
    "orchid1", "black", "#6A3D9A", "blue1", "#CAB2D6", "gray70", "yellow3")
names(pts.colors) <- levels(factor(pts))

#-- Preparing a metadata data.frame that includes:
#-- the sample name
#-- the patient associated to the sample
#-- the technology used to generate the sample
meta.data <- melt(c(names(vars.rna), names(vars.atac)))
row.names(meta.data) <- meta.data$value
meta.data$Patient <- gsub("\\-.*", "", meta.data$value)
meta.data$Method <- paste0(gsub(".*\\_", "", meta.data$value), "-seq")
meta.data$value <- NULL

#-- Setup heatmap annotation - plotData
ha_column = HeatmapAnnotation(df = meta.data[colnames(plotData), "Patient",
    drop = FALSE], col = list(Patient = pts.colors), show_annotation_name = FALSE)
#-- Build a heatmap of the RNA-seq allele frequencies for each variant
hrna <- Heatmap(plotData, col = viridis(100, option = "plasma"), show_row_names = TRUE,
    show_column_names = FALSE, top_annotation = ha_column, column_title = "RNA-seq",
    show_column_dend = FALSE, show_row_dend = FALSE, column_title_gp = gpar(fontsize = 24),
```

```r
    row_names_gp = gpar(fontsize = 2), name = "Allele\nfreq.")

#-- Extract counts of putative candidates from the ATAC-seq
atac.counts <- pullcountsVars(vars.atac, row.names(plotData))
plotData.atac <- atac.counts$M/(atac.counts$M + atac.counts$N)

#-- Setup heatmap annotation for plotData.atac
ha_column = HeatmapAnnotation(df = meta.data[colnames(plotData.atac), "Patient",
    drop = FALSE], col = list(Patient = pts.colors))
#-- Build a heatmap of the ATAC-seq allele frequencies for each variant
hatac <- Heatmap(plotData.atac, col = viridis(100, option = "plasma"),
    show_row_names = TRUE, show_column_names = FALSE, top_annotation = ha_column,
    column_title = "ATAC-seq", show_column_dend = FALSE, show_row_dend = FALSE,
    column_title_gp = gpar(fontsize = 24), row_names_gp = gpar(fontsize = 2),
    name = "Allele\nfreq.")

#-- Plot heatmaps for both technologies side-by-side
hrna + hatac
```

```r
#-- Extract mean allele freq per patient per technology and merge
df.rna <- getptMean(plotData, "RNA-seq")
df.atac <- getptMean(plotData.atac, "ATAC-seq")

#-- Sanity check all data.frames should be similar
all(c(identical(df.rna$Patient, df.atac$Patient)), identical(df.rna$var,
    df.atac$var))

#-- merge into a new data.frame
df.both <- df.rna
df.both$"ATAC-seq" <- df.atac$"ATAC-seq"

#-- Perform a correlation test on the samples with Pearson
cor_both <- cor.test(df.both$ATAC, df.both$RNA)
colnames(df.rna)[2] <- "Patient"
```

```r
#-- Plot the correlation and allele frequency comparison
ggplot(df.both, aes(x = `RNA-seq`, y = `ATAC-seq`, color = Patient)) +
    geom_point(size = 4) + theme_classic(base_size = 14) + geom_abline(slope = 1,
    color = "grey", linetype = "dashed") + scale_colour_manual(values = pts.colors) +
    annotate("text", x = 0.25, y = 0.8, label = paste0("r = ", cor_both$estimate)) +
    ylab("ATAC-seq\nAllele frequency") + xlab("RNA-seq\nAllele frequency") +
    theme(plot.margin = unit(c(1, 1, 1, 1), "cm"), legend.text = element_text(size = 8)) +
    guides(color = guide_legend(ncol = 2))
```

```r
#-- Merge the RNA-seq and ATAC-seq allele frequency data.frames
merged.vars <- merge(plotData.atac, plotData, by = "row.names", all = T)
row.names(merged.vars) <- merged.vars$Row.names
merged.vars$Row.names <- NULL

#-- Create distance matrix from merged method data.frame
#-- The default method is set to 'euclidean'
distmat <- data.frame(as.matrix(dist(t(merged.vars))))
colnames(distmat) <- row.names(distmat)
```

```r
distance.colors <- list(Method = c(`RNA-seq` = "red", `ATAC-seq` = "blue"),
    Patient = pts.colors)
ha_column <- HeatmapAnnotation(df = meta.data[colnames(distmat), ], col = distance.colors)

#-- S8
Heatmap(distmat, col = viridis(100), show_row_names = FALSE, show_column_names = FALSE,
    top_annotation = ha_column, show_column_dend = FALSE, show_row_dend = FALSE,
    name = "Euclidean\ndistance")
```

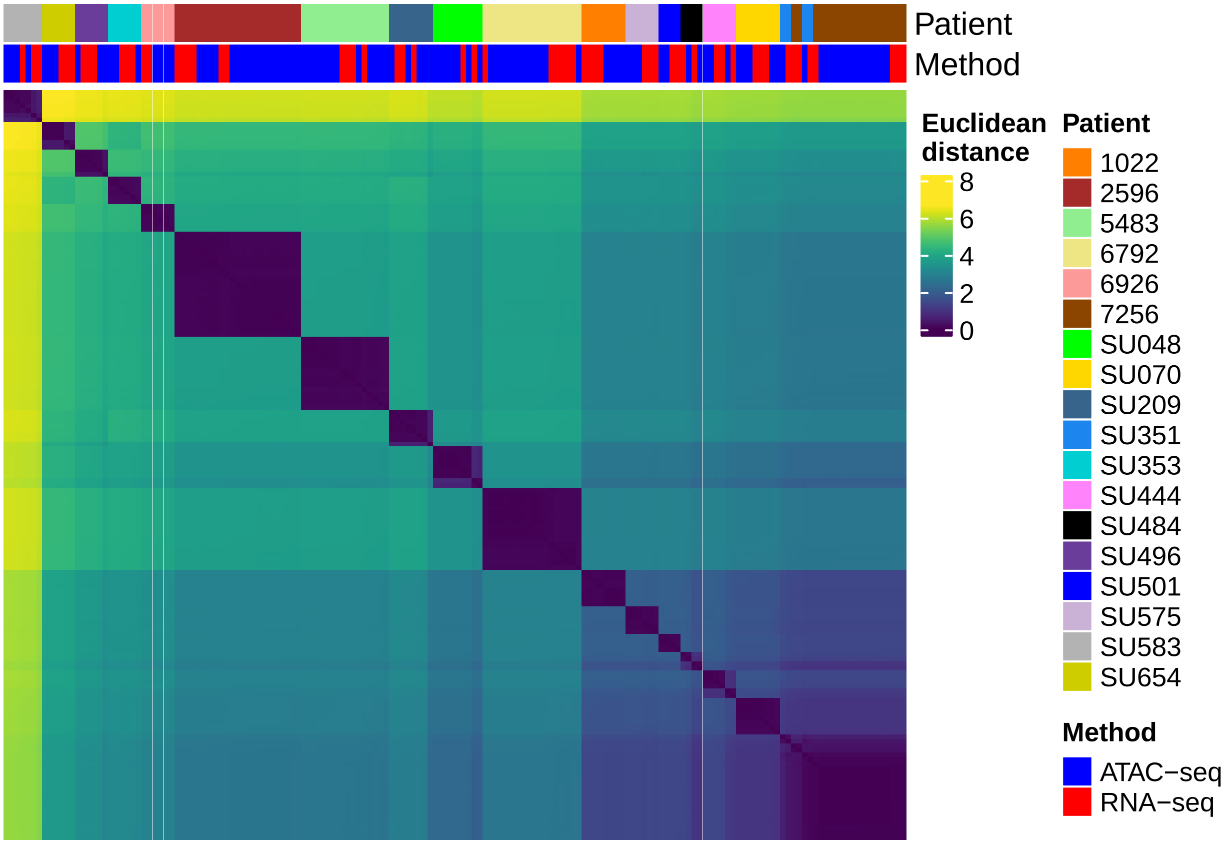

Supplementary Figure S8. Clustered distance matrix of combined variant allele frequencies from ATAC-seq and RNA-seq, using the *Exclusionlist* filtering method.

3.3 The *Exclusionlist* filtering method compared to the *Cohort* method

The Exclusionlist variant filtering approach increased false negative hits and slightly decreased the correlation between RNA-seq and ATAC-seq allele frequencies. It does not work as well as the cohort method for filtering shared variants but still can be used with confidence to identify clones when no other option is available.

In conclusion, we suggest that users always use the cohort method whenever possible (i.e. access to multiple similar datasets).

4 Adding metadata to Seurat objects

Below is just an example where we add the variant allele frequencies calculated above to the minimal test dataset included with Seurat for illustrative purposes.

```
library(Seurat)

## randomly assign our labels to cells
set.seed(123)
colnames(pbmc_small)[seq(colnames(plotData))] <- sample(colnames(plotData))

## add the VAF information
pbmc_small <- AddMetaData(pbmc_small, col.name = "Variant AF", metadata = plotData["1336G>A",
    ])

## plot the default tSNE and the VAF colored one
DimPlot(pbmc_small) + FeaturePlot(pbmc_small, features = "Variant AF",
    cols = viridis(2))
```

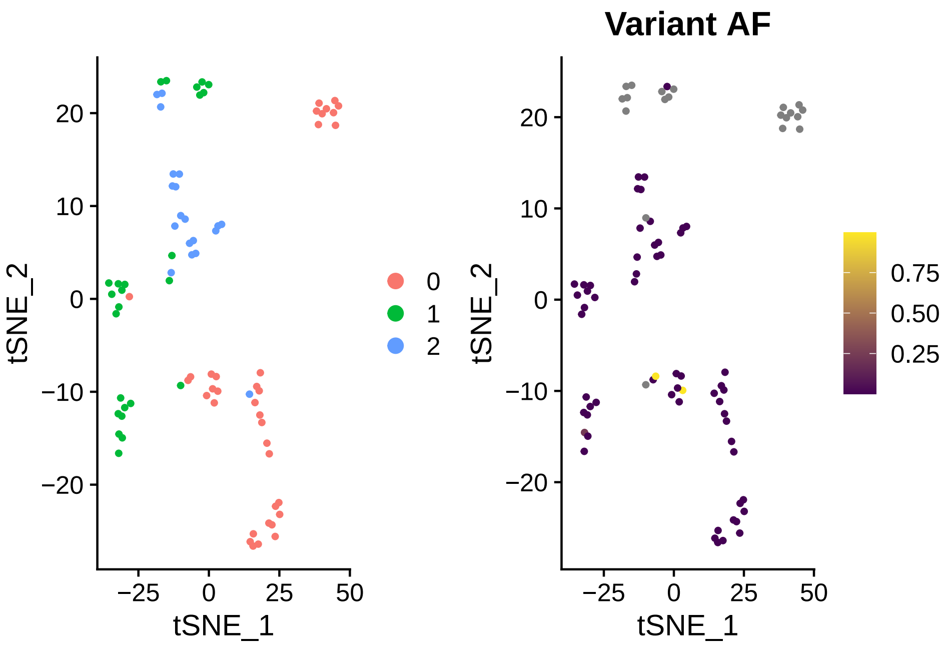

Session information

```
## R version 4.3.2 (2023-10-31)
## Platform: aarch64-apple-darwin20 (64-bit)
## Running under: macOS Sonoma 14.2.1
##
## Matrix products: default
## BLAS:   /Library/Frameworks/R.framework/Versions/4.3-arm64/Resources/lib/libRblas.0.dylib
## LAPACK: /Library/Frameworks/R.framework/Versions/4.3-arm64/Resources/lib/libRlapack.dylib;  LAPACK v
##
## locale:
```

```
## [1] en_US.UTF-8/en_US.UTF-8/en_US.UTF-8/C/en_US.UTF-8/en_US.UTF-8
##
## time zone: Europe/Zurich
## tzcode source: internal
##
## attached base packages:
## [1] grid      stats     graphics  grDevices utils     datasets  methods
## [8] base
##
## other attached packages:
##  [1] Seurat_5.0.1          SeuratObject_5.0.1    sp_2.1-2
##  [4] knitr_1.45            ComplexHeatmap_2.18.0 viridis_0.6.5
##  [7] viridisLite_0.4.2     reshape2_1.4.4        ggplot2_3.5.1
## [10] mitoClone2_1.8.1
##
## loaded via a namespace (and not attached):
##   [1] RcppAnnoy_0.0.21            splines_4.3.2
##   [3] later_1.3.2                 BiocIO_1.12.0
##   [5] bitops_1.0-7                filelock_1.0.3
##   [7] tibble_3.2.1                polyclip_1.10-6
##   [9] XML_3.99-0.16               fastDummies_1.7.3
##  [11] lifecycle_1.0.4             doParallel_1.0.17
##  [13] globals_0.16.2              deepSNV_1.48.0
##  [15] lattice_0.22-5              MASS_7.3-60
##  [17] magrittr_2.0.3              plotly_4.10.3
##  [19] rmarkdown_2.25              yaml_2.3.8
##  [21] httpuv_1.6.13               sctransform_0.4.1
##  [23] Rhtslib_2.4.0               spam_2.10-0
##  [25] spatstat.sparse_3.0-3       reticulate_1.34.0
##  [27] pbapply_1.7-2               cowplot_1.1.2
##  [29] DBI_1.2.0                   RColorBrewer_1.1-3
##  [31] abind_1.4-5                 zlibbioc_1.48.0
##  [33] Rtsne_0.17                  GenomicRanges_1.54.1
##  [35] purrr_1.0.2                 BiocGenerics_0.48.1
##  [37] RCurl_1.98-1.14             VariantAnnotation_1.48.1
##  [39] rappdirs_0.3.3              circlize_0.4.15
##  [41] GenomeInfoDbData_1.2.11     IRanges_2.36.0
##  [43] S4Vectors_0.40.2            ggrepel_0.9.4
##  [45] irlba_2.3.5.1               spatstat.utils_3.0-4
##  [47] listenv_0.9.0               goftest_1.2-3
##  [49] RSpectra_0.16-1             spatstat.random_3.2-2
##  [51] fitdistrplus_1.1-11         parallelly_1.36.0
##  [53] leiden_0.4.3.1              codetools_0.2-19
##  [55] DelayedArray_0.28.0         xml2_1.3.6
##  [57] tidyselect_1.2.0            shape_1.4.6
##  [59] farver_2.1.1                spatstat.explore_3.2-5
##  [61] matrixStats_1.2.0           stats4_4.3.2
##  [63] BiocFileCache_2.10.1        GenomicAlignments_1.38.0
##  [65] jsonlite_1.8.8              GetoptLong_1.0.5
##  [67] ellipsis_0.3.2              progressr_0.14.0
##  [69] ggridges_0.5.5              survival_3.5-7
##  [71] iterators_1.0.14            foreach_1.5.2
##  [73] tools_4.3.2                 progress_1.2.3
##  [75] ica_1.0-3                   Rcpp_1.0.12
```

```
##  [77] glue_1.7.0                  gridExtra_2.3
##  [79] SparseArray_1.2.3           xfun_0.41
##  [81] MatrixGenerics_1.14.0       GenomeInfoDb_1.38.5
##  [83] dplyr_1.1.4                 withr_2.5.2
##  [85] formatR_1.14                fastmap_1.1.1
##  [87] fansi_1.0.6                 digest_0.6.33
##  [89] R6_2.5.1                    mime_0.12
##  [91] colorspace_2.1-0            scattermore_1.2
##  [93] tensor_1.5                  spatstat.data_3.0-3
##  [95] biomaRt_2.58.0              RSQLite_2.3.4
##  [97] tidyr_1.3.0                 utf8_1.2.4
##  [99] generics_0.1.3              data.table_1.14.10
## [101] rtracklayer_1.62.0          htmlwidgets_1.6.4
## [103] prettyunits_1.2.0           httr_1.4.7
## [105] S4Arrays_1.2.0              uwot_0.1.16
## [107] pkgconfig_2.0.3             gtable_0.3.4
## [109] blob_1.2.4                  lmtest_0.9-40
## [111] XVector_0.42.0              htmltools_0.5.7
## [113] dotCall64_1.1-1             clue_0.3-65
## [115] scales_1.3.0                Biobase_2.62.0
## [117] png_0.1-8                   rstudioapi_0.15.0
## [119] rjson_0.2.21                nlme_3.1-164
## [121] curl_5.2.0                  cachem_1.0.8
## [123] zoo_1.8-12                  GlobalOptions_0.1.2
## [125] stringr_1.5.1               KernSmooth_2.23-22
## [127] parallel_4.3.2              miniUI_0.1.1.1
## [129] AnnotationDbi_1.64.1        restfulr_0.0.15
## [131] pillar_1.9.0                vctrs_0.6.5
## [133] RANN_2.6.1                  VGAM_1.1-9
## [135] promises_1.2.1              dbplyr_2.4.0
## [137] xtable_1.8-4                cluster_2.1.6
## [139] evaluate_0.23               GenomicFeatures_1.54.1
## [141] cli_3.6.2                   compiler_4.3.2
## [143] Rsamtools_2.18.0            rlang_1.1.2
## [145] crayon_1.5.2                future.apply_1.11.1
## [147] labeling_0.4.3              plyr_1.8.9
## [149] stringi_1.8.3               deldir_2.0-2
## [151] BiocParallel_1.36.0         munsell_0.5.0
## [153] Biostrings_2.70.1           lazyeval_0.2.2
## [155] spatstat.geom_3.2-7         Matrix_1.6-4
## [157] RcppHNSW_0.5.0              BSgenome_1.70.1
## [159] patchwork_1.2.0             hms_1.1.3
## [161] bit64_4.0.5                 future_1.33.1
## [163] KEGGREST_1.42.0             shiny_1.8.0
## [165] SummarizedExperiment_1.32.0 highr_0.10
## [167] ROCR_1.0-11                 igraph_2.0.3
## [169] memoise_2.0.1               bit_4.0.5
```

References

Dobin, A., Davis, C. A., Schlesinger, F., Drenkow, J., Zaleski, C., Jha, S., Batut, P., Chaisson, M., and Gingeras, T. R. (2013). Star: ultrafast universal rna-seq aligner. *Bioinformatics*, **29**(1), 15–21.

Garrison, E. and Marth, G. (2012). Haplotype-based variant detection from short-read sequencing. *arXiv preprint arXiv:1207.3907*.

Granja, J. M., Klemm, S., McGinnis, L. M., Kathiria, A. S., Mezger, A., Corces, M. R., Parks, B., Gars, E., Liedtke, M., Zheng, G. X. Y., Chang, H. Y., Majeti, R., and Greenleaf, W. J. (2019). Single-cell multiomic analysis identifies regulatory programs in mixed-phenotype acute leukemia. *Nature Biotechnology*.

Kwok, A., Qiao, C., Huang, R., Sham, M.-H., Ho, J., and Huang, Y. (2022). Mquad enables clonal substructure discovery using single cell mitochondrial variants. *Nat Commun*, **13**, 1205.

Lareau, C., Ludwig, L., Muus, C., Gohil, S., Zhao, T., Chiang, Z., Pelka, K., Verboon, J., Luo, W., Christian, E., Rosebrock, D., Getz, G., Boland, G., Chen, F., Buenrostro, J., Hacohen, N., Wu, C., Aryee, M., Regev, A., and Sankaran, V. (2021). Massively parallel single-cell mitochondrial dna genotyping and chromatin profiling. *Nat Biotechnol*, **39**, 451–461.

Li, H. (2011). A statistical framework for snp calling, mutation discovery, association mapping and population genetical parameter estimation from sequencing data. *Bioinformatics*, **27**, 2987–2993.

Ludwig, L., Lareau, C., Ulirsch, J., Christian, E., Muus, C., Li, L., Pelka, K., Ge, W., Oren, Y., Brack, A., Law, T., Rodman, C., Chen, J., Boland, G., Hacohen, N., Rozenblatt-Rosen, O., Aryee, M., Buenrostro, J., Regev, A., and Sankaran, V. (2019). Lineage tracing in humans enabled by mitochondrial mutations and single-cell genomics. *Cell*, **176**, 1325–1339.e22.

McKenna, A., Hanna, M., Banks, E., Sivachenko, A., Cibulskis, K., Kernytsky, A., Garimella, K., Altshuler, D., Gabriel, S., Daly, M., and DePristo, M. (2010). The genome analysis toolkit: A mapreduce framework for analyzing next-generation dna sequencing data. *Genome Res*, **20**, 1297–1303.

Velten, L., Story, B., Hernández-Malmierca, P., Raffel, S., Leonce, D., Milbank, J., Paulsen, M., Demir, A., Szu-Tu, C., Frömel, R., Lutz, C., Nowak, D., Jann, J.-C., Pabst, C., Boch, T., Hofmann, W.-K., Müller-Tidow, C., Trumpp, A., Haas, S., and Steinmetz, L. (2021). Identification of leukemic and pre-leukemic stem cells by clonal tracking from single-cell transcriptomics. *Nat Commun*, **12**, 1366.
